# A Crossover Trial of a Novel Toothbrushing Method for Prevention of Aspiration Pneumonia: Toothpaste With Povidone-Iodine and Moisturizing Gel Mixture

**DOI:** 10.7759/cureus.75494

**Published:** 2024-12-10

**Authors:** Madoka Funahara, Akira Imakiire, Ryuichiro Funahara, Haruka Oyama, Sakiko Soutome, Atsuko Nakamichi

**Affiliations:** 1 School of Oral Health Sciences, Faculty of Dentistry, Kyushu Dental University, Kitakyushu, JPN; 2 Department of Oral Health, Nagasaki University Graduate School of Biomedical Sciences, Nagasaki, JPN; 3 Department of Dentistry and Oral Surgery, Geriatric Dentistry, Funahara Dental Clinic, Hyogo, JPN; 4 Department of Dental Hygiene, Funahara Dental Clinic, Hyogo, JPN

**Keywords:** moisture gel, number of bacteria, povidone-iodine, saliva, toothbrushing

## Abstract

Introduction: Toothbrushing, during which dental plaque is brushed off into the oral cavity, can increase the risk of aspiration pneumonia in older adults and intubated patients.

Methods: This study examined brushing methods to prevent the spread of bacteria in the oral cavity. Six participants who required assistance with brushing received toothbrushing from a dental hygienist. Toothbrushing was performed using a toothbrush soaked in water (Water group), gel (Gel group), povidone-iodine solution (PV-I group), or a mixture of a moisturizing gel and povidone-iodine gel (PV-I+Gel group). The number of bacteria in the saliva before and after brushing was measured using a delayed real-time polymerase chain reaction, which can quantify the number of viable bacteria.

Results: In the Water group, salivary bacterial counts increased significantly after brushing. The bacterial counts in the Gel and PV-I groups increased slightly after brushing; however, the increase was less than that observed in the Water group. In the PV-I+Gel group, the number of bacteria in the saliva was significantly reduced after brushing.

Conclusions: In patients at risk for aspiration pneumonia, toothbrushing should be performed with a mixture of PV-I and a moisturizing gel. This method is a novel approach that reduces the risk of aspiration pneumonia in intubated patients and older adults requiring care, and its clinical application is expected in the future.

## Introduction

Older adults individuals are at a heightened risk for pneumonia, which remains the leading cause of mortality in this age group. The majority of pneumonia cases in older adults are attributed to aspiration pneumonia, primarily due to decreased swallowing function, an increased likelihood of aspirating saliva containing pathogenic microorganisms, weakened immune function, and multiple chronic comorbidities [1]. Aspiration pneumonia poses significant risks not only for the elderly but also for perioperative patients undergoing invasive surgical procedures. Ventilator-associated pneumonia (VAP) in patients requiring endotracheal intubation is believed to be caused by the aspiration of bacteria such as *Staphylococcus aureus*, *Streptococcus pneumoniae*, and gram-negative bacilli mixed with saliva and other oral secretions [2-5]. Both aspiration pneumonia and VAP are primarily triggered by the aspiration of saliva and other fluids containing microorganisms, with the risk increasing proportionally to the number of microorganisms aspirated. While tooth brushing is an effective method for preventing dental caries and periodontal disease, it is also widely employed as an oral care method for frail elderly individuals and perioperative patients [6]. However, it has been reported that brushing can disperse firmly adhered dental plaque into the oral cavity, leading to an increase in bacterial counts in saliva [7]. In healthy individuals, rinsing the mouth with water immediately after brushing and expectorating saliva mitigates the transient increase in bacterial counts. In situations where rinsing is not possible, oral cleansing or suctioning is performed, but these methods cannot completely retrieve all bacteria dispersed in the oral cavity [7]. Therefore, we believe that brushing should be avoided in frail elderly individuals and perioperative patients, particularly those who are intubated, from the perspective of preventing aspiration pneumonia.

Conversely, abstaining from brushing for extended periods raises concerns about the exacerbation of dental caries and periodontal disease and an increased risk of distant infections from oral foci. Thus, establishing a tooth-brushing method that does not elevate the risk of aspiration pneumonia is desired. Attempts have been made to prevent the dispersion of plaque by using moisturizing gels in conjunction with brushing [8]. Additionally, while 0.12% chlorhexidine, widely used abroad for VAP prevention, is prohibited for oral use in Japan, reports suggest that povidone-iodine is an effective antiseptic alternative [9,10]. The present study aims to investigate whether the combination of moisturizing gel and povidone-iodine can suppress the dispersion of viable bacteria from plaque during brushing.

## Materials and methods

Study design and participants

This crossover study was reported in accordance with the Consolidated Standards of Reporting Trials (CONSORT, 2010). The study included older adults and adults with disabilities who were unable to brush independently, specifically those who were capable of making decisions and agreed to participate. The exclusion criteria were iodine allergy and the inability to spit saliva samples. The participants’ sex, age, number of remaining teeth, and Oral Hygiene Index-Debris Index (OHI-DI) were recorded. This preliminary study included six participants. The enrollment date for the first participant was February 12, 2024, and the study was conducted over a period of four weeks with a one-week washout period between each method.

Intervention

**Figure 1:**
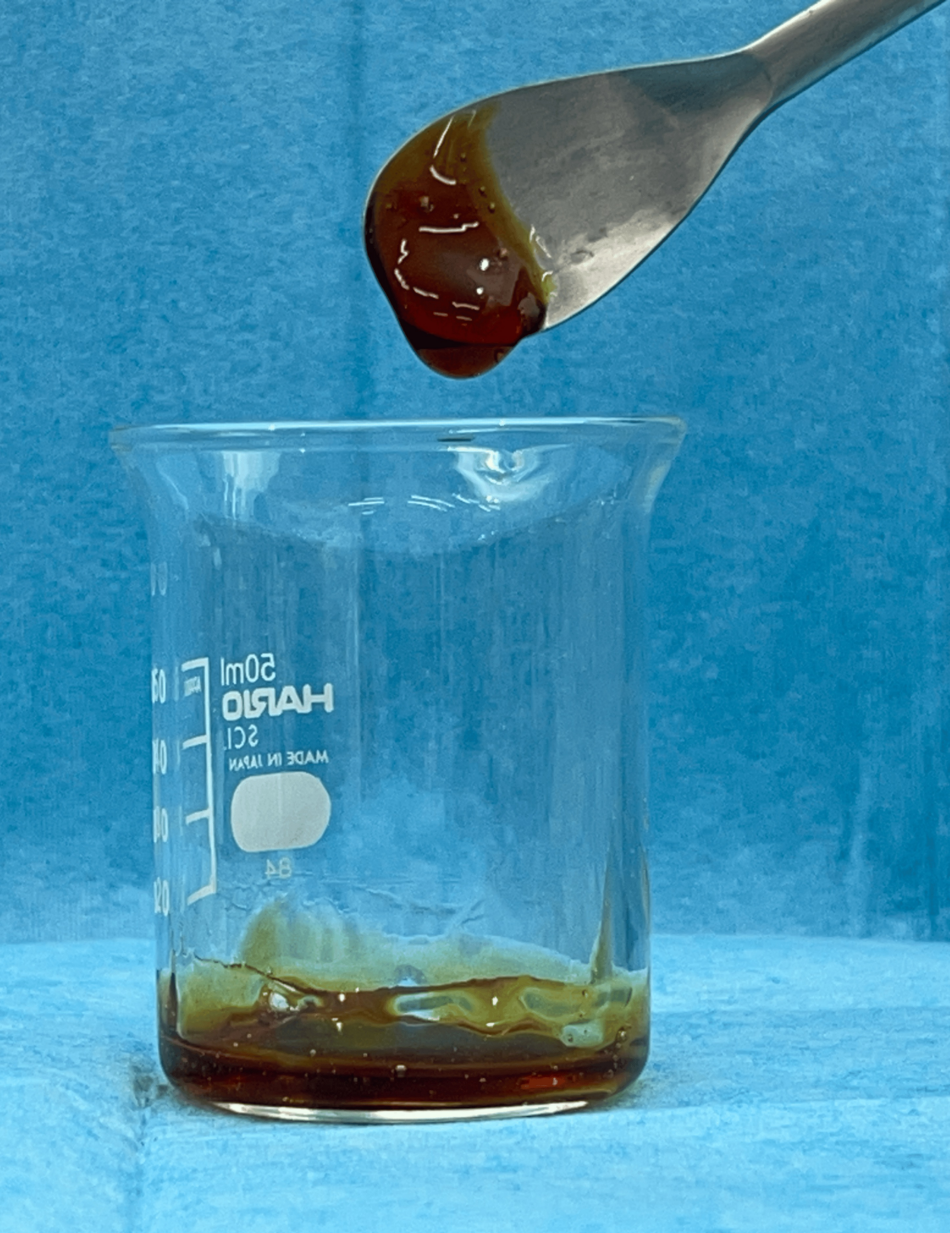
PV-I+gel. A mixture of povidone-iodine gel and moisturizing gel in a 1:9 ratio.

Initially, OHI-DI scores [11] were measured. Dental hygienists administered one of the four brushing methods to the participants. Each brushing was performed using a new toothbrush. Saliva samples were collected before and immediately after brushing. After a seven-day washout period, the three brushing methods were administered sequentially. The brushing methods were as follows. (1) Water group (n=6): The toothbrush bristles were soaked in tap water to moisten them, and every quadrant was brushed for 15 seconds. The toothbrush bristles were then cleaned and soaked in tap water again, followed by brushing. (2) Gel group (n=6): Four grams of oral care gel (OKUCHI WO ARAU GEL, Nippon Shika Yakuhin Co., Ltd., Yamaguchi, Japan) was placed on the toothbrush bristles, and every quadrant was brushed for 15 seconds. The toothbrush bristles were washed, and brushing was repeated for every quadrant after repeatedly washing the toothbrush bristles and placing the gel on them. During brushing, a sponge brush was used to remove contaminated gel containing dental plaque. (3) Povidone-iodine solution (PV-I) group (n=6): The toothbrush bristles were dipped in a povidone-iodine mouthwash solution diluted to the concentration required for mouthwashing according to the manufacturer's instructions (concentration at the time of use: 0.47%), and every quadrant was brushed for 15 seconds. Similarly, the toothbrush bristles were rinsed after brushing each quadrant and dipped in a povidone-iodine solution (PV-I), and brushing was performed again. (4) Moisturizing gel and povidone-iodine gel (PV-I+Gel) group (n=6): Four grams of an admixture of povidone-iodine gel and moisturizing gel in a respective 1:9 ratio were placed on the toothbrush (Figure 1), and every quadrant was brushed for 15 seconds. The toothbrush bristles were washed, and brushing was repeated in every quadrant after repeated washing and placement of the gel on the toothbrush bristles. During brushing, a sponge brush was used to remove contaminated gel containing dental plaque.

Measurement of salivary bacterial counts

The number of bacteria in the saliva was determined using delayed real-time polymerase chain reaction (DR-PCR) [12], which measures the number of viable bacteria in a mixture of viable and dead bacteria. Saliva samples were collected from each group before and after brushing their teeth. A mixed sample containing viable and dead bacteria was prepared. As for dead bacteria, 7% povidone-iodine was added to the saliva sample to dilute 15-fold, left for one minute, and centrifuged at 600 rpm and 4°C for two minutes. After removing the supernatant, the same amount of distilled water was added, and this was repeated twice to create a solution from which povidone-iodine was removed. Dead samples were incubated under anaerobic conditions on brain heart infusion (BHI) agar (Becton, Dickinson and Company, Franklin Lakes, USA) for 48 hours and showed no bacterial growth. Samples with viable bacteria/dead bacteria ratios of 10:0, 5:5, 1:9, and 0:10 were prepared. One hundred microliters of the mixed samples of viable and dead bacteria were incubated in 3 mL of BHI liquid medium for four hours. A standard line was prepared, with the vertical axis representing the growth rate and the horizontal axis representing the logarithm of the percentage of viable bacteria. The percentage of viable bacteria was calculated by fitting the PCR results to a standard curve. To obtain the number of viable bacteria, the percentage of viable bacteria was multiplied by the number of bacteria determined by real-time PCR before liquid culture.

Statistical analysis

All statistical analyses were performed using SPSS v.26.0 (IBM Japan, Tokyo, Japan). The differences in the logarithm of bacterial abundance before and after brushing were analyzed using the Mann-Whitney U test. A p-value <0.05 was considered statistically significant.

Ethics approval

This study conformed to the ethical guidelines of the Declaration of Helsinki and the Ethical Guidelines for Medical and Health Research Involving Human Subjects published by the Ministry of Health, Labor and Welfare of Japan. Ethical approval was obtained from the Institutional Review Board of Kyushu Dental University (no: 23-38, Supplemental file). The study protocol was registered in the University Hospital Medical Information Network (February 5, 2024; JPRN-UMIN000053543).

## Results

**Figure 2:**
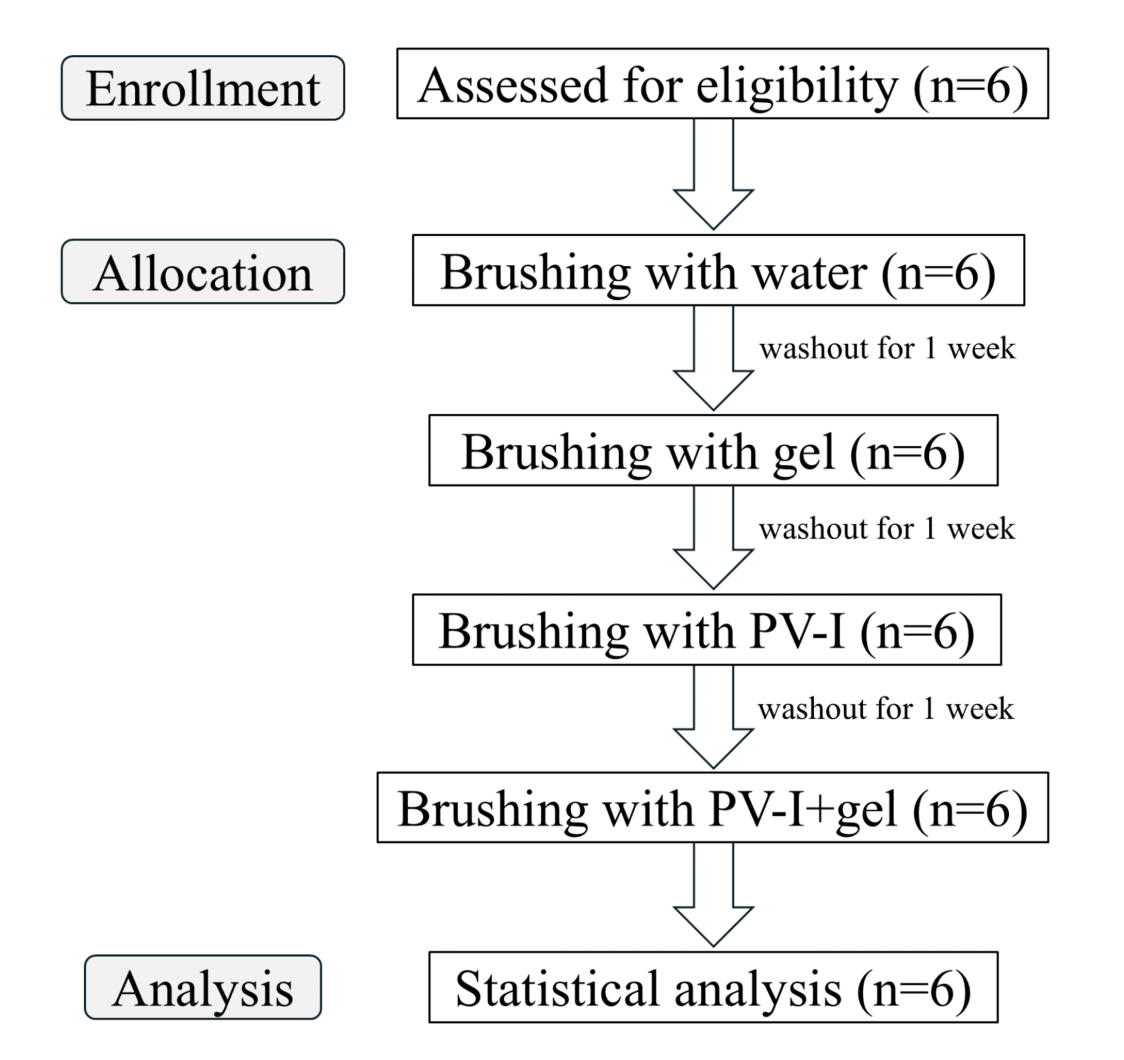
CONSORT diagram. CONSORT: Consolidated Standards of Reporting Trials.

Figure 2 shows the flow chart of this study. Table 1 shows the background characteristics of the six participants. There were four males and two females, with a mean age of 51.83 ± 15.11 years. The mean residual index was 27.17 teeth, and the mean OHI-DI was 3.02 for the Water group, 2.74 for the Gel group, 3.04 for the PV-I group, and 3.00 for the PV-I+Gel group. The six subjects in this study were older adults and adults with disabilities who were unable to brush, but none had diabetes or were being treated with corticosteroids.

**Table 1:** Participant characteristics. Background factors for six volunteer participants. OHI-DI: Oral Hygiene Index-Debris Index, PV-I: povidone-iodine; SD: standard deviation.

Variable	Number of participants/mean ± SD
Sex	
Male	4
Female	2
Age	51.83 ± 15.11
Number of teeth	27.17 ± 2.32
OHI-DI for each group	
Water	1.51 ± 0.22
Gel	1.37 ± 0.25
PV-I	1.52 ± 0.20
PV-I + Gel	1.50 ± 0.50

**Figure 3:**
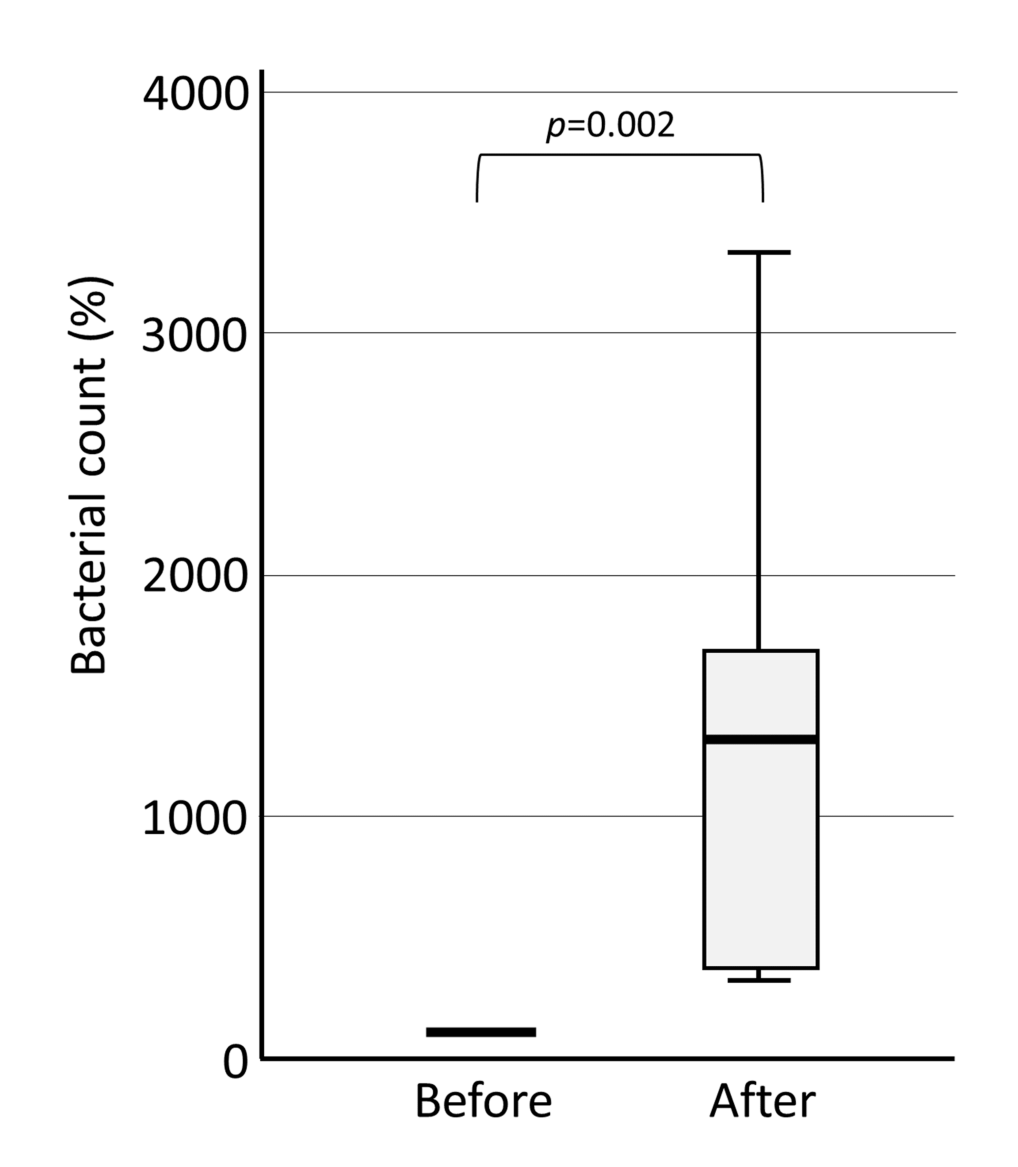
Bacterial count in saliva before and after brushing: Water group. In the Water group, the number of bacteria in saliva was significantly higher after brushing compared to before brushing.

The change in the logarithm of the number of viable bacteria in each group before and after brushing was calculated by setting the pre-brushing value to 100%. In the Water group, the number of viable bacteria increased significantly after brushing, probably due to dental plaque spread by brushing (median 1324%, p=0.002) (Figure 3).

**Figure 4:**
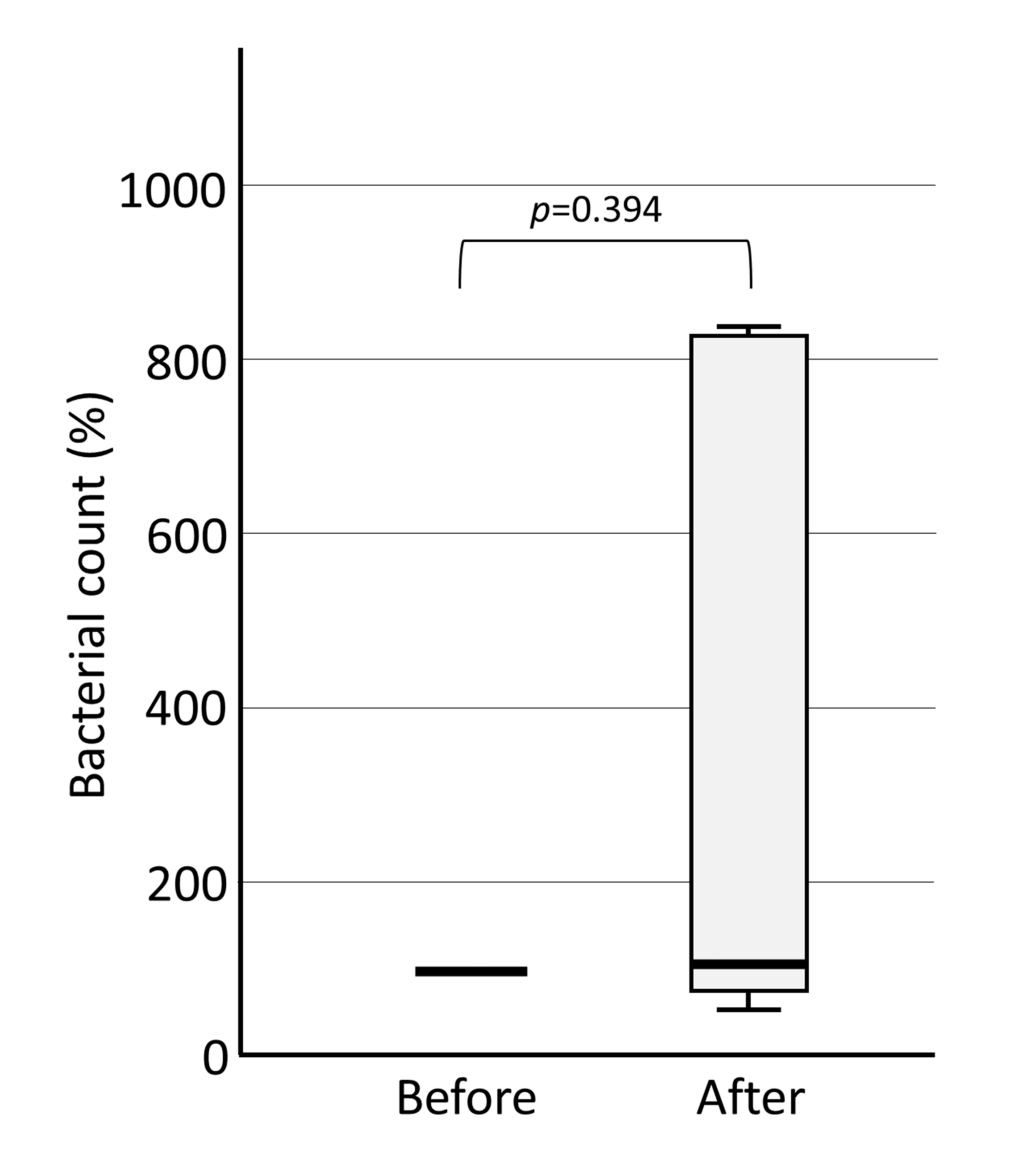
Bacterial count in saliva before and after brushing: Gel group. In the Gel group, there was no significant difference in the number of bacteria in saliva after brushing compared to before brushing.

In the Gel group, the bacterial number slightly increased (median 106.5%). Still, there was a large degree of variation, and no significant difference was found (p=0.394) (Figure 4). The gel group showed a smaller increase in the number of bacteria in saliva than the water group, probably because the gel physically prevented the spread of dental plaque.

**Figure 5:**
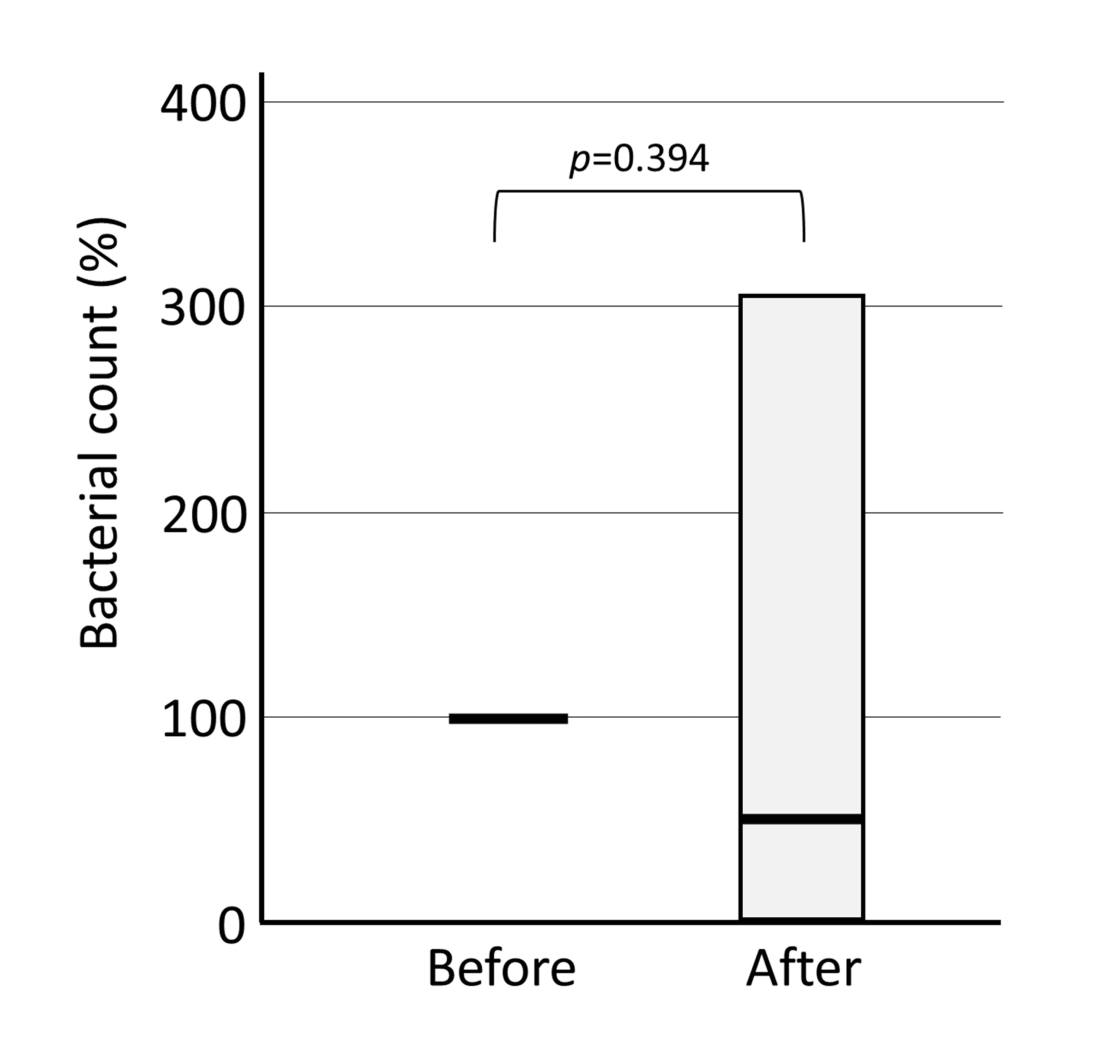
Bacterial count in saliva before and after brushing: PV-I group. In the PV-I group, there was no significant difference in the number of bacteria in saliva after brushing compared to before brushing. PV-I: povidone-iodine.

In the PV-I group, the number of bacteria after brushing varied greatly, and while the median decreased to 52.4%, there was no significant difference (p=0.394) (Figure 5). Although bacteria in the plaque were dispersed during brushing, they were eradicated by the effect of PV-I.

**Figure 6:**
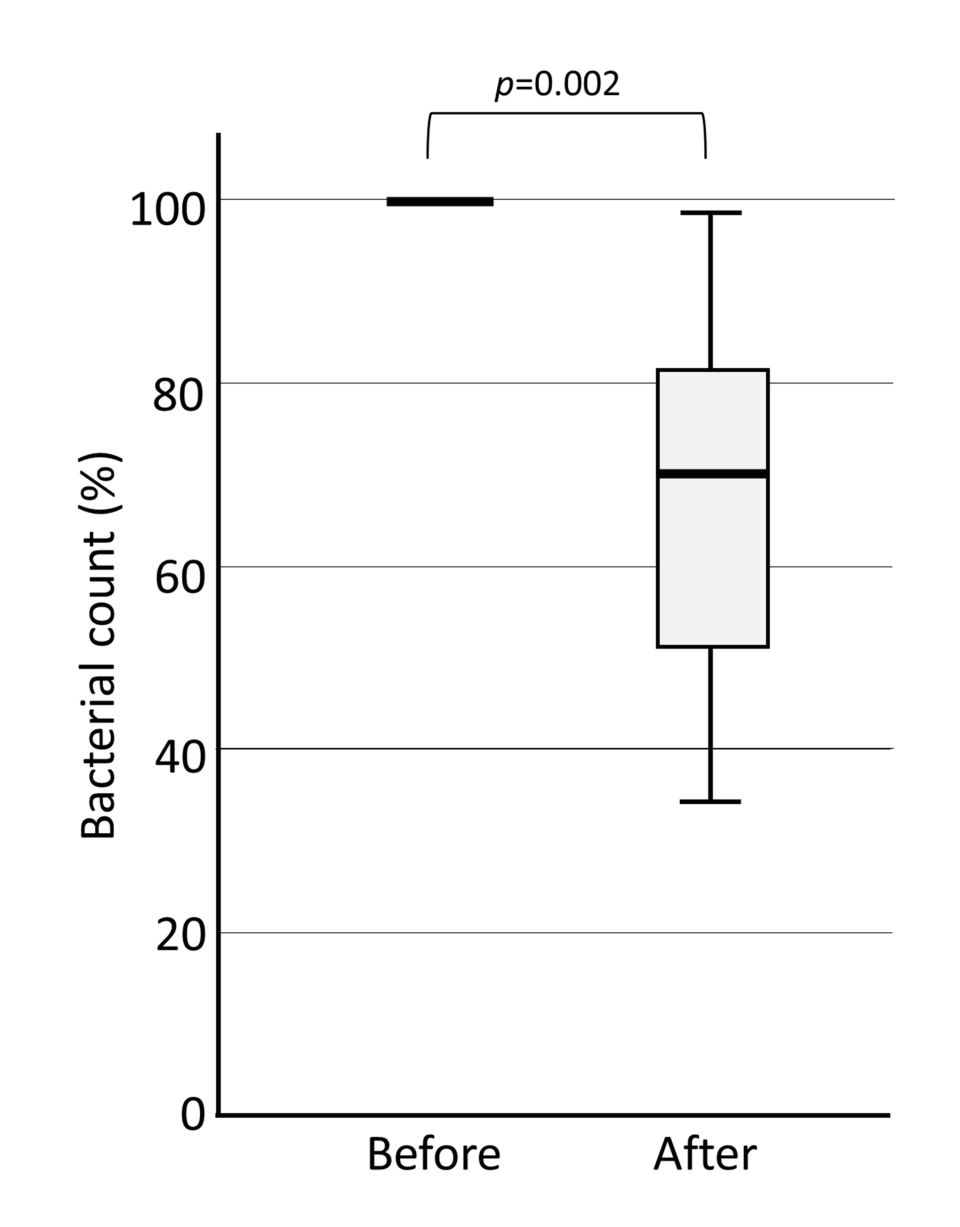
Bacterial count in saliva before and after brushing: PV-I+Gel group. In the PV-I+Gel group, the number of bacteria in saliva was significantly lower after brushing compared to before brushing. PV-I: povidone-iodine.

In contrast, the PV-I+Gel group showed a significant decrease (median 70.4%) from the pre-brushing level (p=0.002) (Figure 6). The physical prevention of plaque dispersion by the gel, combined with the bactericidal effect of the PV-I gel, resulted in a decrease in the bacterial count.

## Discussion

One of the causes of aspiration pneumonia is the aspiration of salivary bacteria. Therefore, it is believed that reducing the number of bacteria in the saliva can prevent aspiration pneumonia, and various approaches to oral care are used. Because the bacteria detected in the foci of pneumonia match the bacteria identified in dental plaque, some experts believe that the causative agent of aspiration pneumonia is the bacteria in the plaque or periodontal pocket, and many reports suggest that this bacteria should be removed to prevent aspiration pneumonia [13,14]. However, the hypothesis that dental plaque causes aspiration pneumonia is questionable because aspiration pneumonia can also occur in edentulous individuals without plaque or periodontal disease.

In a large interventional study of mechanically intubated patients, brushing did not decrease the incidence of VAP [15-17]. Currently, the oral application of 0.12% chlorhexidine is recommended by the Institute of Healthcare Improvement (IHI) ventilator bundle as an oral care method for the prevention of VAP; however, brushing is not included in this bundle [18]. Although some studies have shown that dental plaque removal, such as brushing and professional mechanical tooth cleaning, reduces aspiration pneumonia in older adult patients requiring nursing care [19], other studies have shown that brushing-centered oral care does not reduce the risk of aspiration pneumonia [20,21]. Although reducing the number of bacteria in the saliva is essential for preventing aspiration pneumonia, there is no consensus on whether reducing dental plaque by brushing decreases the incidence of pneumonia. Brushing mechanically removes the plaque that is firmly adherent to the teeth, resulting in the spread of bacteria in the oral cavity. We reported that salivary bacterial counts increased markedly during brushing, the increase could not be suppressed even with wiping, and that the bacterial counts decreased when gargling was performed [7]. These findings suggest that brushing itself may pose a risk of aspiration pneumonia in intubated patients and older adult patients requiring nursing care who are unable to gargle.

On the other hand, if brushing is not performed in older adult patients who require nursing care and in patients who are intubated for long periods, this not only exacerbates dental caries and periodontal disease and worsens oral health conditions such as oral pain, swelling, and drainage, but may also cause infections in organs remote from these oral infection sites. Therefore, as brushing is essential for these patients, we conducted this study because we believe that a brushing method that does not increase the risk of aspiration pneumonia must be established.

Miyahara et al. attempted to prevent the spread of plaque during brushing using a gel in combination with a toothbrush, such that the gel trapped the plaque exfoliated from the tooth surface [8]. In this study, the number of bacteria in the saliva increased markedly after brushing with water; however, the rate of increase tended to be suppressed when a gel was used in combination with water. As PV-I is a potent bactericidal agent, we examined the effect of brushing with PV-I on the inactivation of bacteria by contact when plaque is removed from the tooth surface and allowed to diffuse into the oral cavity. The results showed that PV-I suppressed the increase in the number of salivary bacteria to some extent after brushing. Moreover, the number of bacteria in the saliva was significantly reduced after brushing with a mixture of gel and PV-I, reflecting both the ability of the gel to remove plaque and the chemical bactericidal action of PV-I. The decrease in salivary bacteria may be due to the gel's action and the fact that a portion of PV-I penetrated the saliva and exerted a bactericidal action. These results suggest that brushing with PV-I+Gel is recommended for patients at a high risk of aspiration pneumonia.

This preliminary study had several limitations, including a small sample size and the potential influence of a single researcher's brushing technique and duration. Additionally, the effectiveness of this method under varying oral conditions, such as hygiene and dryness, remains unclear. However, this study demonstrated for the first time that the number of bacteria in saliva increases markedly during brushing and that this increase can be suppressed using the PV-I+Gel. These findings may greatly contribute to the development of oral care methods for older adults requiring nursing care and intubated patients. Future studies will address these limitations by evaluating the method's applicability across diverse patient populations and conditions, thereby establishing its clinical significance.

## Conclusions

Tooth brushing in older adults or patients with disabilities who are unable to rinse may transfer bacteria from dental plaque into the oral cavity, heightening the risk of aspiration pneumonia. In this study, we examined the changes in salivary bacterial counts before and after four different brushing methods. Brushing with water and a toothbrush caused a significant increase in salivary bacterial counts, as bacteria from dental plaque were transferred into the saliva. Brushing with gel and brushing with PV-I resulted in an increase in bacterial counts, but no significant difference was observed. Brushing with a mixture of PV-I+Gel and moisturizing gel led to a significant reduction in viable bacteria after brushing. Therefore, brushing with a mixture of PV-I and gel is considered a safe and effective method for patients unable to rinse, as it prevents the dispersion of plaque while simultaneously providing antibacterial action.
